# What's next for genomics and prion diseases?

**DOI:** 10.4155/fsoa-2017-0021

**Published:** 2017-03-22

**Authors:** Stephanie A Booth

**Affiliations:** 1 Department of Medical Microbiology & Infectious Diseases, College of Medicine, Faculty of Health Sciences, University of Manitoba, Winnipeg, Manitoba, R3E 0J9, Canada; 2 Zoonotic Diseases & Special Pathogens, National Microbiology Laboratory, Canadian Science Centre for Human & Animal Health, Public Health Agency of Canada, Winnipeg, Manitoba, R3E 3R2, Canada

**Keywords:** bioinformatics, genomics, neurodegenerative diseases, Prions

Prions are transmissible agents composed of aggregates of misfolded cellular prion protein (PrP) that cause fatal neurodegenerative diseases. It is increasingly recognized that the mechanism of seeded propagation of aggregates of misfolded proteins is a fundamental molecular process that is common to all of the major human neurodegenerative diseases [1]. Prion diseases arise in one of three ways: via inherited, acquired or sporadic (unknown) causes. Inherited prion disease is associated with mutations in the coding sequence of the prion protein gene (*PRNP*), which lead to misfolding of PrP. More than 30 such mutations have been described to date. Sporadic Creutzfeldt–Jakob disease (CJD) is the most common form of the disease in humans and occurs at an annual incidence of approximately one per million. The initial prompt that sets in motion PrP misfolding is unknown in these cases and may either represent a random event or reflect a somatic mutation in one or more brain cells. Acquired prion diseases include variant CJD (vCJD) that is likely caused by zoonotic transmission via ingestion of foods contaminated with bovine spongiform encephalopathy. Other examples are iatrogenic transmission occurring accidently during medical procedures such as dura mater transplantation or cadaver-derived growth hormone therapy, as well as the human disease kuru that was restricted to isolated populations in Papua New Guinea where the disease was acquired through ritualistic endocannibalism. All types of prion disease are highly heterogeneous in terms of their clinical presentation, the age of onset and in the case of acquired disease, the incubation time.

Studies have already pinpointed a major genetic factor that determines why some people are more susceptible to prion diseases than others. This is a single nucleotide polymorphism (SNP) at codon 129 of the *PRNP* gene that can either be methionine (M) or valine (V). Heterozygosity (M/V) confers relative protection against acquired, sporadic and some inherited forms of CJD either by resistance to the disease or a lengthening of the incubation period [2]. In fact, all cases of vCJD that have been confirmed to date have occurred in patients with the M/M genotype at *PRNP* codon 129. However, one possible case of a patient with heterozygosity was described recently, perhaps suggesting that heterozygosity does lead to an increase in survival [3]. Further evidence to support this exists from observations on kuru transmission. Although kuru has a mean incubation period of around 12 years, some clinical cases are known to have developed as long as 50 years after initial exposure. Retrospective DNA sequencing has revealed that those patients with short incubation periods were for the most part homozygous for M/M or V/V, while recent cases seen in elderly patients who contracted the disease more than 40 years after the funerary feasts were outlawed, were M/V heterozygotes [4]. Notably, another *PRNP* sequence variant, G127V, was recently identified, which confers strong protection in heterozygote individuals that were unaffected but exposed to kuru. Indeed, this amino acid substitution seems to be as protective as deletion of the whole prion gene, rendering the protein refractory to conversion and acting as an inhibitor of prion propagation [5]. Interestingly, the 127V polymorphism was found only in New Guinea populations with high incidences of kuru, yet not at all in kuru patients or unaffected populations worldwide. The appearance of this variant is an example of positive evolutionary selection during the kuru epidemic.

Similar to other neurodegenerative diseases such as Parkinson's and Alzheimer's, prion diseases are now considered multifactorial involving numerous synergistic genetic and environmental factors. Patients develop diseases at different ages and show significantly diverse symptoms, therefore non-*PRNP* genetic factors likely contribute to the development of disease, although very few genes have been identified to date. Studies in inbred lines of mice led to the discovery of a handful of quantitative trait loci that are responsible for the length of the incubation time [6]. Estimates from these studies suggest that approximately 50% of the variance in incubation period is determined by genetic factors other than the *PRNP* gene itself. The exact identities of these genes have yet to be uncovered and are the focus of ongoing genetic studies using new genomic technologies.

Genome wide association studies (GWAS) predict genetic associations by statistical association of the frequency of sequence variants between patients and the population as a whole. This generally requires the analysis of tens of thousands of individuals to produce sufficient statistical power to detect variants with small effects. Human prion diseases occur in only 1 per million of the population; consequently genomic studies of patients have identified only a very small number of genetic factors that are weakly linked to risk and pathobiology [7–9]. It is likely that a proportion of the genetic predisposition to prion diseases will be explained by highly penetrant variants that are very rare, and these will not be captured in such small patient sample sizes. However, compelling data from GWAS in more common neurodegenerative diseases, such as Alzheimer's and Parkinson's, is uncovering the identity of many genes that are linked to more than one disease entity. Given that there are many similarities between different neurodegenerative diseases including the core pathological event of misfolding and aggregation of host proteins, as well as downstream molecular events, such as overactive neuronal signaling, early loss of synapses and dendritic spine complexity and the activation of glia, this is perhaps to be expected. Therefore, this core group of genes may well prove to also contain risk factors for prion diseases, and studies can be designed to identify these in patients. Determination of such shared mechanistic denominators would be an important step forward in improving our understanding of the underlying pathophysiology of the neurodegenerative process that will undoubtedly lead to new avenues for prevention and treatment.

New technologies for massively parallel sequencing that have been developed over the last decade are revolutionizing the approach to determine genetic variants related to disease risk. Large scale sequencing of exomes (the 1% of the genome that encodes proteins) is already routine in many laboratories, and even whole genome sequencing of individuals is rapidly becoming a cost-effective option for high-throughput genomic analysis of patients. The human genome project has led to excellent annotation of the protein coding component of the genome and so genetic variants that alter amino acid sequences can be experimentally validated for altered functionality. However, this is not true for the remaining 99% of the genome whose function is poorly defined and understood. This region does however contain significant sequence variation that may ultimately contribute to disease. For example, a recent study to determine the variation by whole genome sequencing of five patients with a pathogenic mutation for inherited CJD, and 145 healthy individuals, resulted in the observation of 18,648,850 candidate variations, of which only 1102 were within annotated exons [10]. Non-coding regions contain regulatory elements, such as promoters, enhancer's, silencers, locus control regions and insulators that control gene expression by binding of protein factors and DNA methylation, as well as untranslated regions of mRNA to which post-transcriptional regulators such as microRNAs bind [11]. Genetic changes including duplications, deletions, SNPs and translocations in regions outside of the proximal promoters have already been described in both Mendelian diseases, and multifactorial diseases such as diabetes and rheumatoid arthritis. Moreover, GWAS studies in numerous neurological conditions suggest that variations in the levels of gene expression that are attributable to altered regulation do indeed contribute significantly to the genetic risk of disease. Given that mouse models in which the prion protein is overexpressed tend to spontaneously develop disease in later life, it is plausible that dysregulation of the expression of prion protein, and its binding and processing partners, could contribute to the genetic risk of disease. Unraveling the contribution of non-coding regions of the genome has yet to be studied in regards to the development of prion diseases.

Unbiased transcriptional profiling is yet another application of massively parallel sequencing technologies that has yet to be applied to understanding prion disease pathogenesis. Given the limited availability of human brain samples suitable for extraction of RNA, such approaches are generally performed in animal models. Challenges for the future are to tease apart the molecular systems that are activated during disease in the complex network of cells within the brain. Studies to date have not been able to determine the casual relationship between prion misfolding and the damage and death of neurons that results in clinical disease. Transcriptional analysis in single cells as they undergo pathological changes will ultimately be required to define the molecular pathways that are involved. In addition, determination of protein expression, post-translation modifications and protein–protein interactions need to be considered. Analysis of proteins from single cells is in its infancy but the development of such techniques will be especially important. High-throughput single cell sequencing may also be able to answer one of the outstanding questions in prion biology, whether the development of sporadic forms of CJD can be explained by somatic mutation in brain cells [12].

Structural Genomics, a new discipline focusing on the determination of structures of large numbers of proteins without prior knowledge of their function, is of mounting importance in understanding the causal relationship between prion misfolding and the initiation of neurodegeneration. Major advances in determining the elusive structure of an infectious prion have been made in the last few years, in particular by the use of high-resolution cryoelectron microscopy. These new structural determinations may well be pivotal for understanding the fundamental process of protein misfolding as it pertains to prion replication and prion neurotoxicity [13,14]. Prion structures, used in combination with high throughput scanning of chemical structure databases, can be used to model interactions thus enabling the prediction of active prion-drug complexes. The anticipated payoffs of structural genomics will be to accelerate the development of new families of drugs directly targeted at prion replication and neurotoxicity.

Without doubt we are entering an era in which unprecedented amounts of data are being collected routinely from both genetic studies and a vast array of experimental platforms. The challenge is to create informatics tools with which to integrate these layers of data. Network analysis and visualizations are already making inroads to the identification of essential molecules and modules in these biological systems. Continuing the development of new computational tools to mine and interpret the avalanche of ‘omics’ data, that is, currently being generated is undoubtedly the major challenge of the next decades.
